# The impact of Gender Identity Clinic waiting times on the mental health of transitioning individuals

**DOI:** 10.1192/j.eurpsy.2022.2205

**Published:** 2022-09-01

**Authors:** N. Henderson, V. Selwyn, J. Beezhold, R. Howard, R. Gilmore, I. Bartolome

**Affiliations:** 1 University of East Anglia, Norwich Medical School, Norwich, United Kingdom; 2 Norfolk and Norwich University Hospital, Mental Health Liaison Service, Norwich, United Kingdom; 3 University of East Anglia, Psychiatry, Norwich, United Kingdom

**Keywords:** gender, Transgender, Waiting times, Transitioning

## Abstract

**Introduction:**

Waiting times for gender identity services, even before the Covid-19 pandemic, have been a cause of concern. Despite the waiting time standard for planned elective care in the NHS being a maximum of 18 weeks, the average waiting time for a first appointment with a gender identity clinic is 18 months. This study aims to analyse the effect that these timings have on the transgender community, and whether they impact the risk of developing mental health conditions such as depression or anxiety.

**Objectives:**

This study’s main aim is to analyse the correlation between waiting times and mental health burden in the transgender community.

**Methods:**

A literature review and analysis on a transgender individual’s mental health and waiting times for Gender Identity Clinics; looking at any key themes and conclusions. Research papers were taken from MEDLINE, The International Journal of Transgender Health, Oxford Academic, SpringerLink and Emerald Insight, with studies publishing date ranging from 2014 – 2021.

**Results:**

The transgender population were found to have higher rates of suicidal ideation, depression and self harm compared to the general population. Longer waiting times were found to contribute to feelings of low mood and suicidal ideation, as well as decreasing overall quality of life.

**Conclusions:**

Longer waiting times can decrease a transgender individual’s quality of life and impact their overall mental wellbeing: especially with the impact of COVID-19 and the rise in referrals.

**Disclosure:**

No significant relationships.

